# Effect of combined microbes on plant tolerance to Zn–Pb contaminations

**DOI:** 10.1007/s11356-015-5094-2

**Published:** 2015-08-07

**Authors:** Anna Ogar, Łukasz Sobczyk, Katarzyna Turnau

**Affiliations:** Plant-Microbial Interaction Research Group, Institute of Environmental Sciences, Jagiellonian University, Krakow, Poland; Ecosystem Ecology Research Group, Institute of Environmental Sciences, Jagiellonian University, Krakow, Poland; The Malopolska Center of Biotechnology, Jagiellonian University, Krakow, Poland

**Keywords:** Plant/microbial interactions, Arbuscular mycorrhizal fungi (AMF), N_2_-fixing bacteria, Cyanobacteria, Co-cropping, Plant vitality

## Abstract

The presence and composition of soil microbial communities has been shown to have a large impact on plant–plant interactions and consequently plant diversity and composition. The goal of the present study was to evaluate impact of arbuscular mycorrhizal fungi (AMF) and nitrogen-fixing bacteria, which constitutes an essential link between the soil and the plant’s roots. A greenhouse pot experiment was conducted to evaluate the feasibility of using selected microbes to improve *Hieracium pilosella* and *Medicago sativa* growth on Zn–Pb-rich site. Results of studies revealed that biomass, the dry mass of shoots and roots, increased significantly when plants were inoculated with mycorrhizal fungi and nitrogen-fixing bacteria. The addition of *Azospirillum* sp. and *Nostoc edaphicum* without mycorrhiza suppressed plant growth. Single bacterial inoculation alone does not have a positive effect on *M. sativa* growth, while co-inoculation with AMF improved plant growth. Plant vitality (expressed by the performance index) was improved by the addition of microbes. However, our results indicated that even dry heat sterilization of the substratum created imbalanced relationships between soil-plant and plants and associated microorganisms. The studies indicated that AMF and N_2_-fixers can improve revegetation of heavy metal-rich industrial sites, if the selection of interacting symbionts is properly conducted.

**Electronic supplementary material:**

The online version of this article (doi:10.1007/s11356-015-5094-2) contains supplementary material, which is available to authorized users.

## Introduction

Environmental problems arising from tailings containing heavy metals are windblown dust dispersal, leaching of contaminants into surface and groundwaters. Phytoremediation strategies aims to decrease the environmental impact from the heavy metal laden waste by establishing vegetation cover over the degraded area. However, plant growth is often inhibited due to metal toxicity and a combination of factors including low nutrient levels, acidity or alkalinity, poor water holding capacity, and poor physical structure. Unfavorable air–water conditions lead to wind erosion in dry periods as well as soil erosion during rainfall (Bradl 2004; Turnau et al. 2012). Plants and plant-associated microbes are involved in many biogeochemical processes operating in the rhizosphere. Plants themselves alter soil chemistry through changes in pH and redox conditions (Alford et al. 2010). They also release various secondary metabolites including inorganic and organic compounds that contribute to nutrient acquisition, accelerating metal mobility or immobilization (Bais et al. 2006; Toljander et al. 2007). Plants are naturally associated with microorganisms whose microbial communities can directly or indirectly affect metal mobility, availability, and uptake of elements. Microbial consortia including mycorrhizal fungi and nitrogen-fixing bacteria could facilitate the survival of their host plants growing on metal-contaminated sites, by producing growth-stimulating substances and/or by conferring increased tolerance to stress (Doornbos et al. 2012; Rajkumar et al. 2012). It has been shown that co-inoculation with arbuscular mycorrhizal fungi (AMF) and nitrogen-fixing bacteria is a promising approach to favor the establishment and survival of legume plants in poor soils (Tsimilli-Michael et al. 2000; Lin et al. 2007; Franzini et al. 2010). However, little is known about the effects of mixed microbial consortia on the establishment of legumes under heavy metal stress conditions. The aim of this study was to screen microbial interactions, which would take place in the rhizosphere of selected plant species grown on Zn–Pb-rich substrate. *Hieracium* taxon was chosen as a model plant genus since it occurs in a large variety of habitats and the genus have a worldwide distribution. In open environments, *Hieracium pilosella* shows vigorous clonal growth, resulting in the formation of dense mats. The leguminous species *Medicago sativa* has been suggested as a good candidate for remediation of metal-rich tailings primarily due to its ability to fix atmospheric N_2_ and increase the pH of acid soils (Gardea-Torresdey et al. 1998; Mar Vázquez et al. 2000; Turnau et al. 2012). The remediation research has been carried out in Zn–Pb Trzebionka tailing since 1990 (Turnau et al. 2012). The study employed a full factorial experiment on *H. pilosella*, legume *M. sativa*, and microbial communities derived from Zn–Pb-rich tailing. Substrate sterilization procedures have been used to study the effect of introduced microbes on plant growth and to eliminate the influence of other soil microbial communities including soil-borne plant pathogens. Plants were inoculated with combinations of selected microbes such as *Rhizophagus irregularis* (syn. *Glomus intraradices*) and indigenous strains of *Azospirillum* sp. and *Nostoc edaphicum*. The concept of single, dual, and/or multilevel co-inoculations was studied in relation to plant performance. The study was performed to (1) assess the effectiveness of microbial inoculation and co-cropping in the formation of a more stable vegetation cover on Zn–Pb tailings, (2) determine interaction between plants and microbial consortia, (3) determine possible interactions of co-cropped plants: alfalfa (*M. sativa*) and hawkweed (*H. pilosella*) growing together on contaminated site, (4) check whether microbial inoculation could be useful in establishment of plants on the Zn–Pb-rich substratum, (5) select the most efficient combination of microbial inoculation to improve plant growth on tailing, and (6) compare the effect of microbial inoculation on growth and photosynthetic parameters of plants grown on non-sterilized and dry heat sterilized tailing.

## Materials and methods

### Site characterization

The ZG Trzebionka Mine Company is located near Chrzanów (Southern Poland, 30 km west of Kraków, N 50° 09′, E 19° 25′). The Chrzanów district has a long history of metal ore mining. During the twentieth century, extraction of Zn–Pb ores was carried out by the ZG Trzebionka Mine Company. The Trzebionka ore field is located in the SE part of the Silesian–Cracow ore district. Zinc and lead ore were extracted from Mississippi Valley-type mines where deposits were located in Triassic dolomites. These are strata-bound tabular ore bodies with very complex internal structures. The primary ore minerals are sphalerite (ZnS) and galena (PbS) accompanied by iron sulfides, cerussite, smithsonite, and hemimorphite. The wall rock is crystalline dolostone, called “ore-bearing dolomite.” The ore grade is low: 4.2 % Zn and 1.7 % Pb (on average) (Mucha and Szuwarzyński 2004). Since 1970, excavated ores have been subjected to flotation processes. The waste material produced as a by-product of flotation was deposited 1 km from the ore extraction site over ca. 40 years and resulted in the formation of a 60 m high and 64 ha area of tailing. Each year, 2.2 million tons of ores were extracted (Szuwarzyński 1993). Extraction activity was terminated in 2009 when resources were exhausted. The slopes were constructed from the coarser grained fractions of wastes, separated in hydrocyclones (Turnau et al. 2012). The ponds are now a danger to the environment because of the eolian erosion taking place on the surrounding slopes and on the dried out portion of the plateaus above the slopes. Low porosity results in unfavorable air–water conditions, restricted water infiltration during rainfall, and restricted water recharge by capillary rise from deeper layers during dry periods. These conditions favor wind erosion in dry periods and water erosion during rainfall (Trafas 1996). The list of vascular plant species recorded on ZG Trzebionka zinc wastes were presented at Turnau et al. 2012.

### Waste material characterization

The chemical composition of the tailing material is unfavorable for plant growth because the carbonate content exceeds 75 %. High concentrations of Ca^2+^ and SO_4_^2−^ ions together with low concentrations of Na^+^, K^+^, Mg^2+^, Cl^−^, and HCO_3_^−^ ions are also typical for this site. Original tailing material contains no organic matter and is P- and N-deficient. The pH of the waste ranges from 7 to 8. Growth substratum from the Zn–Pb tailing contained high concentrations of heavy metals: 468 μg g^−1^ cadmium, 7068 μg g^−1^ lead, and 53,303 μg g^−1^ zinc. The analysis of metal content in substratum was done using total reflection X-ray fluorescence (TXRF) (Turnau et al. 2008). The soil moisture ranged from 0.06 to 0.15 m^3^ m^−3^ (Ryszka and Turnau 2007).

### AMF inoculation

The AMF inocula used was *R. irregularis* UNIJAG PL. 30/BR 1 obtained from the AMF collection (Jagiellonian University, Kraków). *R. irregularis* was propagated on stock cultures with *Zea mays* for 6 months. Colonized root fragments, mycelium, and a sand–soil mixture containing propagules (ca. 100 propagules g^−1^ of substratum) were used as inoculum.

### Isolations of plant growth-promoting rhizobacteria

Both associated bacteria (*Azospirillum* sp. and *N. edaphicum*) were isolated from native ecotype of *H. pilosella* and *M. sativa* roots/rhizosphere that were collected at the Zn–Pb-rich tailing. The bacterial strains were isolated following protocols for PGPR isolation (Bashan et al. 1993). Fifteen strains of rhizobacteria and five strains of diazotrophic bacteria were obtained after isolation. In in vitro cultures, plants were subcultured with bacterial strains on the modified Strullu–Romand medium (MSR) (Promega Benelux, Leiden, The Netherlands). Cultures were cultivated for 2 months in a growth chamber at 24 °C, under a 12/12 h light regime. Two strains of N_2_-fixers: *Azospirillum* sp. and *N. edaphicum* were selected for further examination. Taxonomic identification was based on cell/colony morphologies using the following references: (Starmach 1966; Ettl and Gärtner 1995; Hoek 1995; Hindák 1996; Jeffery et al. 2010).

#### *Azospirillum* sp.

The bacterial inoculum of *Azospirillum* sp. was grown on nutrient broth (Difco Bacto, USA). Flasks were incubated at 26 °C, for 36 h on a rotary shaker (170 rpm). Actively growing cells were then washed three times with sterile phosphate-buffered saline (100 mM phosphate buffer; 0.85 % NaCl; pH 7.0) by centrifugation (10 min, 15,000*×g*) (Murty and Ladha 1988). The washed cells were resuspended in the same buffered saline as described earlier to a final concentration of about 10^8^ colony-forming units (cfu)/ml. Bacterial suspension with an optical density at 600 nm (OD_600_) corresponded to a final concentration of ∼10^8^ bacteria/ml. Twenty milliliters of the bacterial inoculation suspensions was poured onto the substrate surface of each pot to initiate infection and subsequent nodulation. Control treatments were inoculated with sterilized (121 °C, 20 min, 1 bar) inoculation suspension.

### *Nostoc edaphicum* Kondrateva

Cyanobacteria, *N. edaphium*, were grown in 250-ml Erlenmeyer flasks containing 100 ml of Jaworski medium at pH 7.0. The flasks were incubated at 26 °C, for 3 weeks on a rotary shaker (120 rpm). Actively growing cells were harvested by centrifugation (10 min, 15,000*×g*) then washed three times with sterile physiological saline (9 g l^−1^ NaCl) and resuspended in sterile. Treatments without cyanobacteria were treated in the same way with sterilized (121 °C, 20 min, 1 bar) inoculation suspension. Twenty milliliters of the bacterial inoculation suspensions was poured onto the substrate surface of each pot.

### Experimental design

A greenhouse experiment utilizing a full factorial randomized block design was implemented. The experiment was conducted with the use of two plant species (*H. pilosella* L. and *M. sativa* L.). *H. pilosella* was chosen as a model plant based on observations made on ZG Trzebionka site where this plant grows vigorously. Its main way of spreading is clonal growth. It usually formed new seedlings on the top of the partly dried tufts, and the new ramets were formed outside. Such ramets sometimes disappeared while it was hot and dry, but after the rain, they were usually rebuilt from the remaining parts. In the places where *H. pilosella* appeared in the following seasons, some other accompanying plants established, such as *M. sativa*. The growth of *M. sativa* was nearby *H. pilosella* patches, very rarely growing alone on this particular tailing. Due to N_2_-fixing abilities, *Medicago* species were proposed as good candidates for remediation strategies to enrich poor in nutrient substrata. There were several attempts to introduce *M. sativa*, but this plant was not able to survive there. Therefore, an experiment under laboratory conditions on zinc–lead waste was carried out to discover if the growth of alfalfa can be improved by the co-cropping and introduction of mycorrhizal fungi and N_2_-fixing bacteria.

Control plants *H. pilosella* (Hp) and *M. sativa* (Ms) were grown separately, and they were also co-cropped (Hp + Ms). No inoculation was provided for control plants. Eight different inocula variant were applied: (1) AMF; (2) *Azospiriullum* sp.; (3) *Azospiriullum* sp. + AMF; (4) *Nostoc edaphicum*; (6) *N. edaphicum* + AMF; (7) *Azospirillum* sp. + *N. edaphicum*; (8) *Azospiriullum* sp. + *N. edaphicum* + AMF. Plants were grown on the Zn–Pb-rich tailing. Experiment was conducted on the non-sterile (NS), where no treatments were provided, and on the sterile substratum (S), where dry heat sterilization was applied to sterilized substratum. Waste material was sterilized at 100 °C for 2 h, over 3 days in a row and then allowed to cool for 72 h to eliminate biotic communities, but still retain abiotic tailing traits. Next, the plants were inoculated with arbuscular mycorrhizal fungi *R. irregularis* (M), *Azospirillum* sp. (A), and *N. edaphicum* (N) in different combinations or left non-inoculated (controls). Each combination was replicated five times for a total of 100 pots. Two compartmented cultivation systems with 37-μm polyester mesh (Sefar LFM, Switzerland) were provided to separate *H. pilosella* and *M. sativa* roots. Seeds of *H. pilosella* ecotype were obtained from populations grown at an industrial waste disposal area ZG Trzebionka. Seeds of *M. sativa* (V29814/04/001) were obtained from Malopolska Hodowla Roslin (HBP, Poland, Krakow). Both types of seeds were surface sterilized with (1 % chloramine T (3 min), 6 % sodium hypochlorite (2 min), and 90 % ethanol (2 min)) and washed three times with distilled sterile water between each step. Seeds of *H. pilosella* were pregerminated on 3 % water agar under greenhouse conditions. Fourteen-day-old seedlings were transferred into plastic pots (14 cm tall by 11 cm wide) and filled with 500 g of tailing material. Each pot was sown with 100 seeds of Alfalfa. Plants were watered three times a week with deionized water. Pots were regularly weighed to maintain moisture content at 80 % of water-holding capacity. The plants were not supplied with any nutrient solution. The experiment was conducted in a controlled environment in growth chamber (PaNELTECH, Poland with an in-built TAC Xenta/Vista system) maintained at 24 °C, under a 12/12 h light regime. Maximum photosynthetic photon flux (PPF) was 110 ± 10 μmol (s m^2^)^−1^, and supplementary light was not needed. Relative humidity ranged from 20 to 25 %. Plants were harvested after 17 weeks of growth.

### Plant vitality

Plant vitality was evaluated before harvesting using a Plant Efficiency Analyzer (Hansatech Instruments, UK) estimating chlorophyll *a* fluorescence transient of intact leaves. Measurements were taken on the upper surface of fully expanded leaves. For each soil/microbe combination, three leaves of each *H. pilosella* plant and 15 *M. sativa* plants per pot were measured. The collected data set was used for the JIP test analysis (analysis of O-J-I-P fluorescence transient) (Strasser and Srivastava 1995; Tsimilli-Michael et al. 2000; Strasser et al. 2004). Although indirect, the JIP test allows information about the structure and function of the photosynthetic apparatus (mostly related to PSII) to be obtained. The photosynthetic efficiencies, i.e., the maximum quantum yield of PSIIat *t* = 0, φPo = TR_0_ / ABS = 1 − (F_o_ / F_m_) = F_v_ / F_m_ was measured. The specific flux parameters chosen to be calculated in the present study, all referring to the condition of the sample at time zero, expressed per reaction center (RC) were analyzed: (1) ABS/RC—the average absorption per RC; (2) TR_0_/RC—the specific trapping flux per RC; (3) DI_0_/RC—the dissipated energy flux per RC; (4) ET_0_/RC—the maximal specific flux for electron transport per RC. Parameters were derived from the theory of energy flux from biomembranes (Strasser et al. 2000, 2004). Also, the performance indexes which are products of terms expressing “potentials” for photosynthetic performance where PI_ABS_: performance index (PI) on absorption basis PI_ABS_ = [RC / ABS] [TR_0_ / (ABS − TR_0_] [ET_0_ / (TR_0_ − ET_0_)] and PI_TOTAL_: total PI, measuring the performance up to the PSI end electron acceptors, PI_TOTAL_ = PI_ABS_ [RE_0_ / ET_0_ − RE_0_)] were analyzed. The logarithms of performance indexes at *t* = 0 log (PI_TOTAL_, PI_ABS_) were defined as the total driving force (DF_TOTAL_, DF_ABS_) for photosynthesis of the observed system, created by summing the partial driving forces for each of several energy bifurcations (Strasser et al. 2004).

### Harvest and sample preparation

Each plant was carefully removed from the pot. Loosely adhering waste material was removed from the roots, and larger root pieces remaining in the substrate were manually picked out. Root and aerial part biomass of harvested plant was weighted separately. Total fresh biomass of roots was weighted; after it, one fourth of roots was taken for staining to estimate mycorrhizal colonization. Collected biomass was frozen at −20 °C and then freeze-dried for 72 h, at −55 °C (Christ Beta 1–8 LD plus, SciQuip Ltd., UK). Dry weights of shoots and roots were determined after the freeze-dried procedure.

### Arbuscular mycorrhizal colonization

At harvest, roots were removed from substrate and then gently rinsed with running tap water and then with deionized water (DI). Mycorrhizal root parts were randomly collected and cleared in 10 % KOH for 24 h at room temperature. Subsequently, after careful washing in tap water, the roots were acidified for 1 h in 5 % lactic acid and stained for 24 h at room temperature in 0.05 % aniline blue in lactic acid, in order to visualize the fungal structures inside the roots. Mycorrhizal colonization was estimated in the roots of *H. pilosella* and *M. sativa*. Root colonization by *R. irregularis* was determined according to protocol provided in Mycorrhiza Manual (www2.dijon.inra.fr/mychintec/Protocole/Workshop_Procedures.html). Since experiment was carried out in plant growth chamber, the possibilities of contamination by airborne spores from other AMF was limited. The following parameters were assessed: intensity of the mycorrhizal colonization in the root system (M%), intensity, and arbuscule abundance in the root system (A%). From each pot, approximately 80 cm of fine root fragments were randomly taken for mycorrhizal estimation. Mycorrhizal colonization and arbuscular richness were estimated microscopically in about 60 1-cm-long root fragments per pot.

### *Medicago sativa* nodules

Roots were shaken gently to remove substrate, then washed gently and dried with moist tissue. Nodules were removed and fixed in formaldehyde–acetic acid–ethanol (FAA). The number of nodules was counted per pot. Different nodules: white, pink, brown, hard, and soft were collected form roots.

### Statistical analysis

For biomass, dry mass, and mycorrhizal parameters, means were compared using ANOVA The Tukey’s test was used as a post hoc test when ANOVA showed significance. If assumptions were not met, we used non-parametric tests (Kruskal–Wallis test).

For photosynthetic parameters, first was performed the principal component analysis (PCA) to select variables with the highest loadings for first and second axis. Then, for selected variables, ANOVA analysis was performed. If assumptions were not met, we used non-parametric tests (Kruskal–Wallis test). For PCA, we used CANOCO 5 software (Ter Braak and Šmilauer 2012). For ANOVA and other tests, we used STATISTICA (version 10 StatSoft, 2010) software.

## Results

### Biomass production

**Fig. 1:**
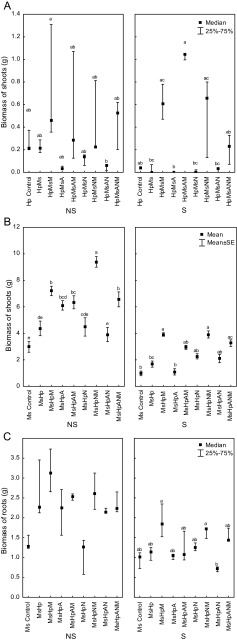
Biomass in (g) of **a** *Hieracium pilosella* shoots (Hp), **b** *Medicago sativa* shoots (Ms), **c** *Medicago sativa* roots; plants grown on *NS* non-sterilized substrate, *S* dry heat sterilized substrate, and inoculated with different microorganisms: *M Rhizophagus irregularis*, *A Azospirillum* sp., *N Nostoc edaphicum*; *different letters above bars* indicate statistically significant differences (*P* < 0.05)

Plants grown (*M. sativa* and *H. pilosella*) in NS Zn–Pb-rich tailing demonstrated stunted growth consistent with S substratum in comparison with plants grown on NS substrate (Fig. 1). Significant differences between microbial treatments regarding biomass were observed. Growth of *H. pilosella* was significantly lower in the dry heat sterilized tailing material than on the non-sterile substratum. Significantly higher (*p* < 0.05) shoots biomass/dry mass was observed when *H. pilosella* was inoculated with microbes. Especially when plants were grown on sterile substrate, AMF inoculation together with diazotrophs: *Azospirillum* sp. and *N. edaphicum* exerted a positive effect on *Hieracium* growth (Fig. 1a). No significant changes of *H. pilosella* biomass were observed in the non-sterile substrate compared to the control plants (Fig. 1a). Although, stimulation of *H. pilosella* growth was observed when plants were co-inoculated with AMF and N_2_-fixers while compared to single inoculation with N_2_-fixers (*Nostoc* or *Azospirillum*) (Fig. 1a). Similar trends were also obtained for root biomass and dry weights (data not shown).

Mycorrhizal inoculation together with N_2_-fixers always stimulated alfalfa growth, while single and double inoculation with *Azospirillum* sp. or *N. edaphicum* did not have a positive effect on *M. sativa* shoot growth (Fig. 1b). In S substrate, single inoculation with *Azosporillum* sp. was as high as in the case of control plants (Fig. 1b). Biomass of *M. sativa* grown on both (S) and (NS) substrates increased, when plants were inoculated with *R. irregularis*. On sterile substrate, positive effect of mycorrhizal inoculation was even more pronounced (Fig. 1b). Root growth of *H. pilosella* was greatly reduced when plants were supplied with a single inoculation with a *N. edaphicum* in NS treatment and double inoculated with both N_2_-fixers in S substrate (Fig. 1c).

To conclude, growth of non-inoculated plants in both substrates (S and NS variant) was reduced. Dry heat sterilization procedure had the greatest effect, reducing shoots and root growth of both plant species. In general, shoot biomasses as well as dry weights (not shown) of *H. pilosella* and *M. sativa* were higher in non-sterile substrate than in sterile one (Fig. 1a, b). Always, presence of AMF positively affected fresh biomass of both tested plant species (*p* < 0.05). There was significant interaction between sterilization procedure and various microbial treatments for *H. pilosella*. Co-inoculation (AMF + N_*2*_-fixers) was effective and increased productivity of *H. pilosella* grown on the Trzebionka tailing. The greatest *M. sativa* shoot development was reached in plants dually inoculated with *R. irregularis* + *N. edaphicum* on both substratum (S and NS). Non-inoculated control plants and inoculated plants did not show any differences in root growth except inoculation with *N. edaphicum* (on non-sterile substrate) and double N_2_-fixer inoculation (on sterile substrate), which negatively affects alfalfa root growth (Fig. 1c).

### Root AMF colonization

#### Intensity of the mycorrhizal colonization in the root system (M%)

**Fig. 2:**
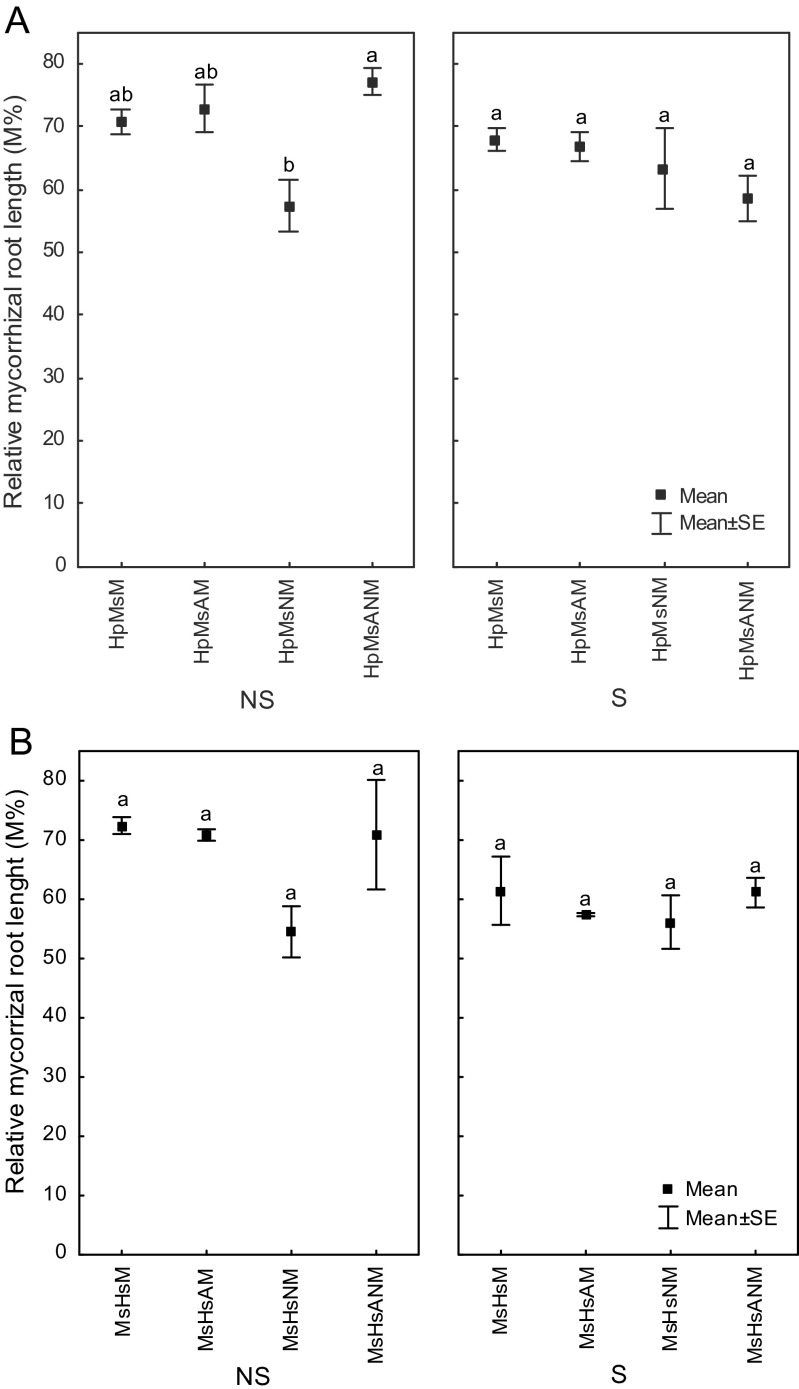
Relative mycorrhizal root length (M%) evaluated for **a** *Hieracium pilosella* (Hp), **b** *Medicago sativa* (Ms), when plants were grown on *NS* non-sterilized substrate, *S* dry heat sterilized substrate; plants were inoculated with *M Rhizophagus irregularis*, *A Azospirillum* sp., *N Nostoc edaphicum*; *different letters beside the values* indicate statistically significant difference (*P* < 0.05)

Roots of inoculated plants were extensively colonized by *R. irregularis*, and percentage root colonization of *M. sativa* was comparable with that of *H. pilosella*. Dry heat sterilization procedure caused decreases of mycorrhizal colonization of both plants (Fig. 2a, b). In the case of *M. sativa*, no statistically important interactions were found between substratum treatment and inoculation option, while significant interactions were observed for *H. pilosella*. The most efficient treatments were those when *H. pilosella* was co-inoculated with *Azospirillum* sp. as well as when double inoculation with both N_2_-fixers was provided (Fig. 2a). Alfalfa inoculated with *N. edaphicum* (N) and *R. irregularis* (M) grown on sterile as well as on non-sterile substratum were significantly different from all other treatments (Fig. 2b). Addition of *N. edaphicum* significantly decreased intensity of the mycorrhizal colonization in the root system of both plant species whether plants were grown on sterilized substrate or not (Fig. 2a, b).

#### Arbuscular richness: arbuscule abundance in the root system (A%)

**Fig. 3:**
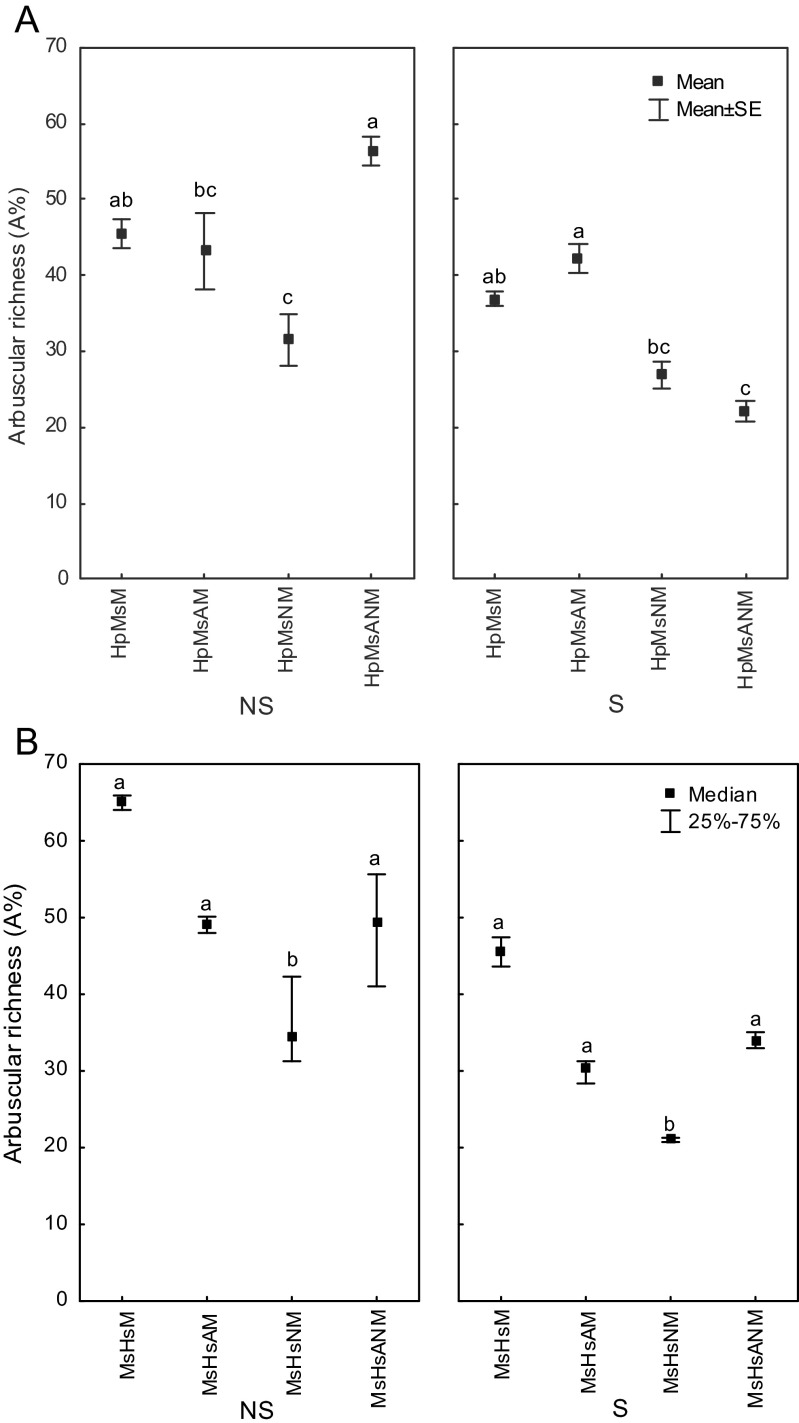
Relative arbuscular richness (A%) evaluated for **a** *Hieracium pilosella* (Hp), **b** *Medicago sativa* (Ms), when plants were grown on *NS* non-sterilized substrate, *S* dry heat sterilized substrate; plants were inoculated with *M Rhizophagus irregularis*, *A Azospirillum* sp., *N Nostoc edaphicum*; *different letters beside the values* indicate statistically significant difference (*P* < 0.05)

On sterilized substrate, mycorrhizal colonization was significantly lower. Significant decreases in A% values were observed for both plant species. Arbuscule richness in root fragments where the arbuscules were abundant (A%) showed important differences between substrate sterilization treatment and microbial inoculation variant for *H. pilosella* species. When *H. pilosella* was grown on non-sterile substrate, the highest rate of arbuscular richness was observed for plants inoculated with both N_2_-fixers (Fig. 3a). While, single inoculation of *H. pilosella* with cyanobacteria, as well as double with *N. edaphicum* and *Azospiruillum* sp. negatively affected arbuscule abundance (A%) in the root system on sterile substrate (Fig. 3a). The arbuscule abundance in the alfalfa root system (A%) was significantly higher when plants were grown on non-sterile substrate when compared to sterile system (Fig. 3b). Inoculation with N_2_-fixers caused decrease in the arbuscule richness in root fragments where the arbuscules were abundant (A%) in the *M. sativa* root system (Fig. 3b). However, in non-sterile substrate, other microbes present in the substrate probably attenuated negative effect of N_2_-fixers on arbuscular richness. Sterilization procedure of the substratum prior to decreased mycorrhizal colonization. Inoculation with N_2_-fixers affected the percentage of AMF colonization. The response of *H. pilosella* was different depending on sterilization procedure of the substrate. When plants were grown on non-sterile substrate, nitrogen-fixing microbial inoculants positively influenced *H. pilosella* root colonization, while on sterile substrate, N_2_-fixers negatively affected arbuscule abundance (Fig. 3a). The lowest survival rate and lowest arbuscule abundance that were found for both plants were single inoculation with *N. edaphicum* was applied (Fig. 3a, b).

### Nodule number

**Fig. 4:**
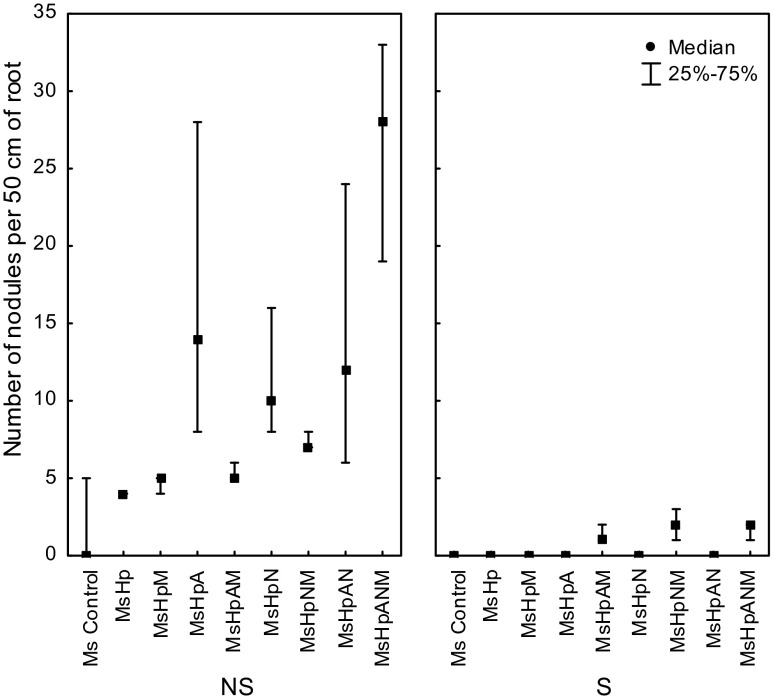
Number of nodules per 50 cm of *Medicago sativa* roots (Ms) co-cropped with *Hieracium pilosella* (Hp). Number of nodules was estimated after 3 months of growth on a Zn–Pb-rich tailing: *NS* non-sterilized substrate, *S* dry heat sterilized substrate. Plants were inoculated with *M Rhizophagus irregularis*, *A Azospirillum* sp., *N Nostoc edaphicum*

The number of nodules in alfalfa inoculated with *R. irregularis* grown on non-sterile were significantly higher, whilst a very few nodules were observed in sterile treatments (Fig. 4). A beneficial effect of applied microbial inoculations was found for both S and NS substrates (Fig. 4). The +AMF treatments significantly enhanced the development of root nodules. Dual inoculation with the *R. irregularis* and the associated diazotrophs (*Azospirillum* sp. and *N. edaphicum*) also resulted in a synergistically increased nodule number. The highest nodule number on non-sterile substrate was found when both N_2_-fixers and AMF was applied (Fig. 4). When *M. sativa* was grown on dry heat sterilized substrate, nodules were formed just when plants were co-inoculated with mycorrhiza and N_2_-fixing bacteria. Otherwise, nodules where not present (Fig. 4).

### Plant vitality

#### *Hieracium pilosella*

PCA analysis showed that there was no differentiation into two plant groups collected from sterile and non-sterile substrate when we consider photosynthetic parameters for *M. sativa*. In the case of *H. pilosella*, two groups of samples were recognized: sterile and non-sterile one (Supplement 1).

**Fig. 5:**
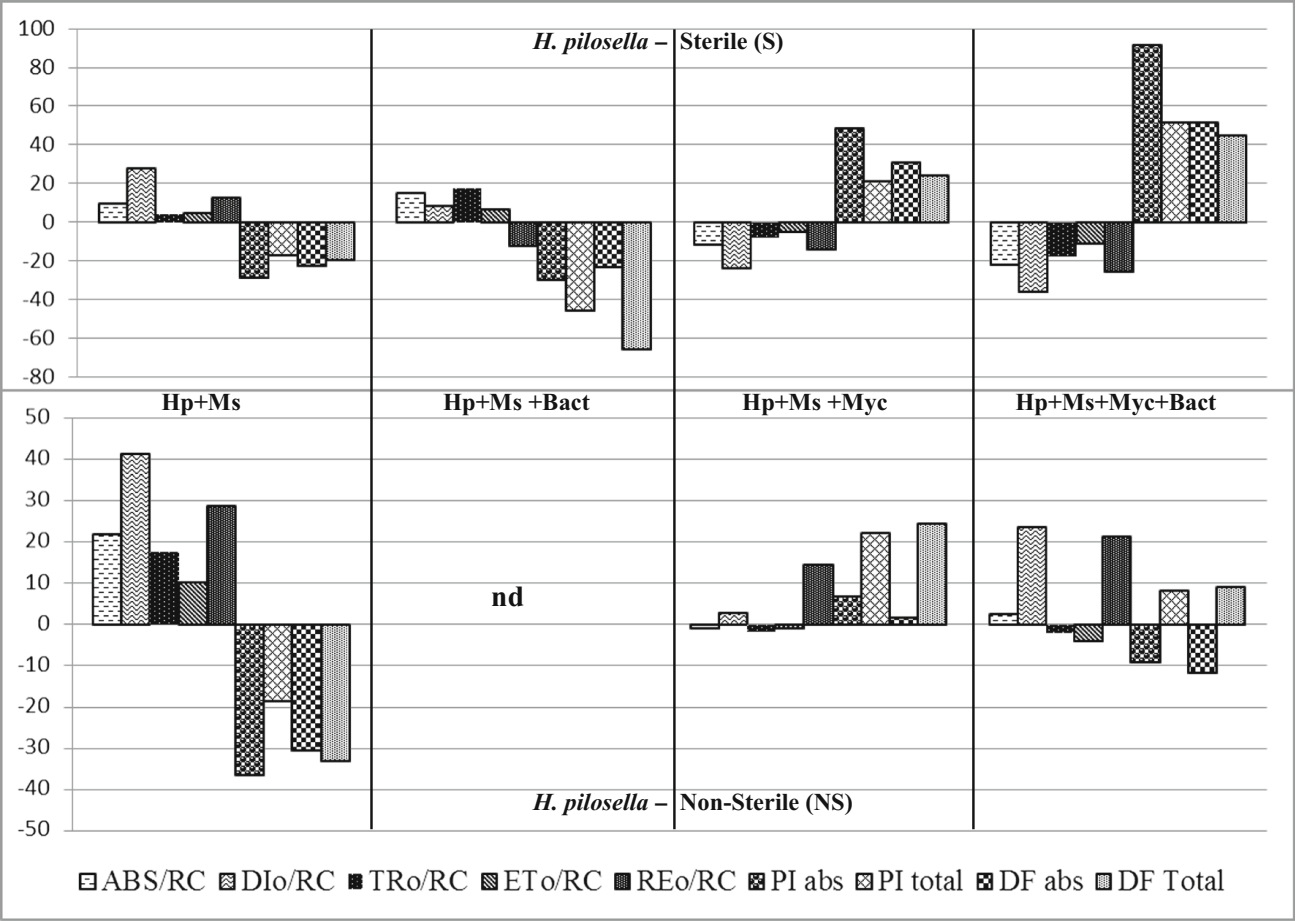
Deviation of the fluxes as expressed relative to control plants *Hieracium pilosella*. The values are expressed in percents (%) and reflect the deviation flux differences between non-inoculated plants and plants after single inoculation with the AM fungus *Rhizophagus irregularis* (Myc), dual inoculation with the diazotrophs bacteria: *Azospirillum* sp. and *Nostoc edaphicum* (Bact), triplicate co-inoculations with AMF fungus *R. irregularis* and diazotroph bacteria (Myc + Bact), plants grown on *NS* non-sterilized substrate, *S* dry heat sterilized substrate. *ABS/RC* the average absorption per RC, *DI*_*0*_ */RC* the dissipated energy flux per RC, *TR*_*0*_ */RC* the specific trapping flux per RC, *ET*_*0*_ */RC* the maximal specific flux for electron transport per RC, *PI*_*ABS*_ performance index on absorption basis, *PI*_*TOTAL*_ total performance index (PI), *DF*_*ABS*_ driving force on absorption basis, *DF*_*TOTAL*_ total driving force

Statistical analysis of photosynthetic parameters obtained for *H. pilosella* showed interaction between plants grown on sterilized substratum/non-sterile and provided inoculation treatment. The result of ANOVA for experimental data showed F_v_/F_m_, TR_0_/RC, ET_0_/RC, RE_0_/RC, and PI_TOTAL_, PI_ABS_, DF_ABS_, and DF_TOTAL_ changed significantly after inoculation (Fig. 5a, b). Microbial inoculation resulted in increase of *F*_v_/*F*_m_ parameter for *H. pilosella* grown on sterile substrate, except the case when *H. pilosella* was only co-cropped with *M. sativa*. Measured values of F_v_/F_m_ were in the range of 0.72–0.75. On non-sterilized substrate, F_v_/F_m_ values were significantly lower (0.67–0.78) only when triplicate co-inoculation was provided (mycorrhiza and both bacterial strains). Single and triplicate AMF inoculation (Myc and Myc + Bact) induced a decrease by 23–10 % of ABS/RC and 38–23 % of DI_0_/RC on sterile substrate; this decrease is counterbalanced in the single or dual inoculation with diazotrophs (Bact). On sterilized substrate, AMF single and triple inoculation (Myc and Myc + Bact) significantly increased PI_ABS_ by (45–90 %) and PI_TOTAL_ by (25–50 %) of *H. pilosella*, while double inoculation with N_2_-fixers caused a decrease of PI parameters (Fig. 5b). Inoculation with AMF and both N_2_-fixers increased DF_ABS_ and DF_TOTAL_ by 25–30 % compared to control (Fig. 5b). On NS substrate, single inoculation with AMF (Myc) and triplicate inoculation with mycorrhiza and diazotrophs (Myc + Bact) decreased both ABS/RC (20 %) and TR_0_/RC parameter (40 %). Figure 5b demonstrates that single inoculation with AMF (Myc) increased PI_ABS_ by 25 %, and the triplicate inoculation with AMF inoculation and both diazotrophs (Myc + Bact) increased PI_TOTAL_ around 10 % (Fig. 5). Single mycorrizal inoculation (Myc) increased DF_TOTAL_ of *H. pilosella* (Fig. 5).

#### *Medicago sativa*

**Fig. 6:**
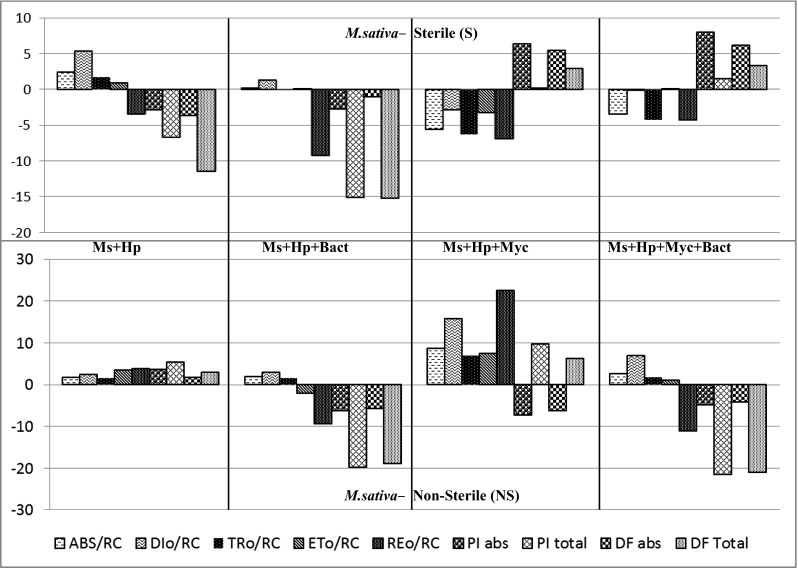
Deviation of the fluxes as expressed relative to control plant *Medicago sativa*. The values are expressed in percents (%) and reflect the deviation flux differences between non-inoculated plants and plants after single inoculation with the AM fungus *Rhizophagus irregularis* (Myc), dual inoculation with the diazotrophs bacteria: *Azospirillum* sp. and *Nostoc edaphicum* (Bact), tripartite co-inoculations with AMF fungus *R. irregularis* and diazotrophs bacteria (Myc + Bact), plants grown on *NS* non-sterilized substrate, *S* dry heat sterilized substrate. *ABS/RC* the average absorption per RC, *DI*_*0*_ */RC* the dissipated energy flux per RC, *TR*_*0*_ */RC* the specific trapping flux per RC, *ET*_*0*_ */RC* the maximal specific flux for electron transport per RC, *PI*_*ABS*_ performance index on absorption basis, *PI*_*TOTAL*_ total performance index (PI), *DF*_*ABS*_ driving force on absorption basis, *DF*_*TOTAL*_ total driving force

The result of ANOVA evaluated for alfalfa showed no interactions observed between sterilization procedure (plants grown on sterile or non-sterile substrate) and provided microbial inoculation. Microbial inoculation did not cause any significant changes of F_v_/F_m_ parameter in alfalfa sample, neither when plants were grown on sterile and non-sterile substrate. F_v_/F_m_ values obtained for alfalfa were in the range of 0.83 and 0.85. The analysis of specific energy fluxes per QA^−^ reducing reaction center revealed differences in absorption (ABS/RC), trapping (TR_0_/RC), reduction of end acceptors at PSI electron acceptor side (ET_0_/RC), and in the dissipated energy flux (DI_0_/RC) between controls and inoculated with microbe plants (Fig. 6). Microbial inoculation affected the performance index PI_ABS_ of alfalfa. PI_ABS_ parameter increased when plants were inoculated with AMF (Myc and Myc + Bact), while bacterial inoculation (Bact) caused a decrease of this parameter (Fig. 6). Figure 6a showed that on sterile substratum, a decrease of ABS/RC, TR_0_/RC, and RE_0_/RC by 4–7 % was observed in the case of single inoculation with AMF (Myc) and triplicate inoculations with mycorrhiza and diazotrophs (Myc + Bact). Different effect was observed for *M. sativa* grown on NS case, where single inoculation with *R. irregularis* (Myc) increased ABS/RC, TR_0_/RC, and RE_0_/RC by 9–22 % (Fig. 6). Although, there was no significant changes in phenomenological energy flux parameters observed in non-sterile treatment when *M. sativa* was double inoculated with diazotrophs (Bact) and triplicate with AMF + N_2_-fixers (Myc + Bact). In NS substrate, just double inoculation with N_2_-fixers (Bact) and triplicate inoculation with AMF + diazotrophs (Myc + Bact) resulted in the decrease of PI_TOTAL_ parameter by 23–20 %. Although, inoculation with mycorrhizal fungi *R. irregularis* (Myc) caused an increase of performance indexes PI_TOTAL_ by 10 %. The same results were observed for DF_TOTAL_ parameter (Fig. 6b).

## Discussion

### Co-cropping

The polluted sites require combination of different restoration approaches such as intercropping, co-cropping, or pre-cropping to improve the establishment of a plant cover on metal-rich industrial wastes (Khan 2005; Sprocati et al. 2014). *H. pilosella* and *M. sativa* were selected because their potential ability to be used in vegetation practices on heavy metal-rich polluted sites. Clonal plants such as *H. pilosella* spread horizontally within their habitat by means of stolons, rhizomes, and ramets. Connection between ramets allow for translocation of resources within the clone. Through a spatial division of labor, clonal species were able to perform specific tasks and closely co-operate by potentially independency including reproduction (Stueffer et al. 1996). Developing vegetative reproduction system and exhibiting phenotypic plasticity allows *H. pilosella* to overcome the establishment risk and regenerate under unfavorable conditions such as dry periods and lack of nutrients (Salzman 1985; Winkler and Stöcklin 2002; Roiloa and Retuerto 2012). *H. pilosell*a populations usually almost exclusively depend on clonal reproduction (Bishop and Davy 1985). It has been claimed that *H. pilosella* is allelopatic species (Murphy and Aarssen 1995; Murphy 2000; Jankowska et al. 2014). However, our experiment reveals suppression of *H. pilosella* by *M. sativa* rather than the reverse. Similar results were obtained for *H. pilosella* and *Arrhenatherum elatius* in colliery spoils of north of France (Henn et al. 1988). The leguminous species *M. sativa* has been suggested as a good candidate for remediation of contaminated soils, due to its rhizobial symbionts and ability to fix atmospheric N_2_ (Gardea-Torresdey et al. 1998, 1999; Peralta-Videa et al. 2002; Lin et al. 2007). Co-cropping different species may enhance the overall capabilities of a phytoremediation. However, under unfavorable conditions like the presence of heavy metals, co-cropping of *H. pilosella* (non-fixers) and *M. sativa* (legume) caused a negative effect regarding *H. pilosella* growth and performance (Figs. 1a and 5a). Environmental conditions may disturb the benefits from co-cropping of non-fixing plants with legumes, which under certain conditions, like a presence of heavy metals, might exhibit a limited capacity to fix nitrogen. On the other hand, potential benefits gained by a neighboring non-fixer plant will strongly depend on their capacity to use effectively the extra N input (Temperton et al. 2007). This could be caused by the competition for other macroelements and microelements as well as for water and light (Tilman et al. 1997).

### Substrate sterilization procedure

In general, different microbial populations may establish themselves under the influence of root exudates and bioavailability of essential nutrients. However, the presence of toxic metals strongly determines the size and composition of microbial populations in the rhizosphere (Bever et al. 2010). Different soil sterilization procedures are proposed in biological researches concerning microbial influence or heavy metal impact on plant growth. Without the assurance of sterile conditions, obtained metal data sets can be misinterpreted as sorption of metals to solid substrate or losses due to biological activities. Of concern is the need to eliminate biological activity in a soil sample where single interactions between plants and selected microorganisms are investigated. Since many sterilization methods can dramatically change physical and chemical properties of the soil, it is therefore important to choose the least destructive. Where chemical stability is required, air-dry sterilization rather than moisture is recommended (Lotrario et al. 1995; Trevors 1996; Egli et al. 2006).

However, our results indicate that even dry heat sterilization disturbed primarily established relationships between soil-plant and plants and associated microorganisms. In our studies, the plants’ response to microbial inoculation was significant when the substrate was dry heat sterilized. In non-sterile substrate, the microbial communities were more complex from the beginning. Already established relationships between native soil microbe communities and roots in non-sterile prevent plants from metal stress. Significantly higher biomass and dry mass production of plants growing on non-sterile substrate indicate better starting conditions. For this reason, additional inoculation of plants growing on non-sterile substrate did not cause such significant plant responses. This sterilization appears to imbalance already established microbial relationships in the soil, which is why we cannot draw the same conclusions from S and non-sterile treatment regarding additional mycorrhizal and N_2_-fixer’s inoculations.

### Arbuscular mycorrhiza interaction with N_2_-fixers

The presence and composition of soil microbial communities have been shown to have large impacts on plant–plant and plant–microbial interactions and consequently plant diversity and composition. The rhizosphere is a specialized niche defined as the root–soil interface, where associated microorganisms, roots, and soil come together (Alford et al. 2010). Root colonization is a competitive process that is affected by environmental conditions such as soil moisture, soil texture and pH, organic matter content, access to the nutrients, as well as specificity of host. Thus, microbial population is one of the essential parts of dynamic rhizosphere system that affect the rhizosphere soil properties such as pH, redox potential, metal concentration, water content, bulk density, root exudation, and all the biological transformations (Barea et al. 2002; Bais et al. 2006; Bakker et al. 2013). However, unfavorable conditions on polluted sites impose the need to compete for nutrients and other resources which are limited. Therefore, individual PGPR strains exhibit specificity in sensitivity to environmental parameters to promote host growth and/or suppress plant pathogens (Hibbing et al. 2010). Depending on the strains used, they may perform a number of tasks ranging from narrow to broad (Doornbos et al. 2012; Bakker et al. 2013). Increasing attention is focused on the interactions between PGPR bacteria and mycorrhizal fungi (Biró et al. 2000; Mar Vázquez et al. 2000; Tsimilli-Michael et al. 2000; Barea et al. 2005a, b). Possible application of AM fungi and different soil bacteria in bioremediation processes like phytostabilization and phytoextraction has been a great concern (Barea et al. 2002, 2005a; Weyens et al. 2009). Studies show that dual inoculation AM fungus with nitrogen-fixing soil bacteria enhanced nitrogen fixation in legumes (Bagyaraj et al. 1979; Biró et al. 2000; Tsimilli-Michael et al. 2000; Scheublin et al. 2004; Temperton et al. 2007). *Azospirillum brasilense* and *Glomus intrarradices* were capable of co-existing in sugar cane roots both intracellularly and intercellularly, causing changes in the cell wall. Sugar cane plant biomass, the number of endophytic microorganisms, and nitrogen-fixing activity increased with joint inoculation (Bellone and de Bellone Silvia 2012). Inoculation with native bacterial and fungal strains ensures best performance under harsh conditions such as high metal content. Beside AMF, diazotrophs, free-living nitrogen-fixing bacteria strongly attributed to nitrogen fixation and cause an increase of nitrogen amount available to plants (Bethlenfalvay et al. 1982; Toro et al. 1998; Biró et al. 2000; Mar Vázquez et al. 2000; Franzini et al. 2010). In case of *M. sativa*, formation of nodules would be the most desirable; however, on Zn–Pb Trzebionka tailings, nodules are rarely found (Turnau et al. 2012), and this was also proved during the present investigation as shown in case of control plants grown on non-sterile substratum. Nodulation is an energetically costly process, and legumes balance the nitrogen demand with the energy expense by limiting the number of nodules (Kassaw et al. 2015). In addition, toxic metals present in the substratum are known to induce morphogenic responses and as a consequence reduce the formation of rhizobial infection (Potters et al. 2007; Lafuente et al. 2010). Those metals can also lower down the expression of several nodulation genes (Lafuente et al. 2010). Bacterial activity and the rhizobial symbiosis are influenced by mycorrhiza, both qualitatively and quantitatively (Barea et al. 2005a, b). Our results shows that additional inoculation with selected microbes increased number of nodules; however, we have to underline that total number of nodules is not as important as number of active one. *H. pilosella* responded positively to mycorrhizal inoculation but not to single inoculation with N_2_-fixing bacterial symbionts. On the other hand, mycorrhizal inoculation together with N_2_-fixers always stimulates alfalfa growth, while single as well as double inoculation with *N. edaphicum* or *Azospirillum* sp. did not have a positive effect on shoot growth. This result may indicate that poor substrate such as Zn–Pb-rich tailings used in our studies may have precluded a net benefit from the mycorrhizal symbiosis more strongly than from N_2_-fixing bacteria. This can be explained by the fact that symbiotic nitrogen fixation is very phosphorus intensive due to the high ATP requirement. AM fungi increased mineral and nutrient uptake where up to 80 % of plant’s phosphorus (P) needs and 25 % of its nitrogen (N) is obtained via the fungus (Marschner and Dell 1994; Smith et al. 2004; Govindarajulu et al. 2005; Parniske 2008). The mycorrhization percentage of plants growing on this particular Zn–Pb tailing was shown to be usually high (Orłowska et al. 2005). While under non-polluted conditions, usually lower percentage of mycorrhization is efficient to obtain plant growth promoting effect (Russo et al. 2005). On industrial tailings, this might be not enough due to involvement of fungal hyphae in sequestering toxic metals, what results in increased metal concentration within roots which is one of the known avoidance mechanisms (Leyval et al. 1997). Overall, mycorrhizal inoculation with AMF always had a positive effect on plant growth. It has been proposed that the presence of their exudates low and high molecular weight compounds including carbohydrates and organic acids. This may create a favorable environment around the mycorrhizal fungal hyphae and cell surface structures, which could have supported microbial growth (Scheublin et al. 2004; Toljander et al. 2007; Miransari 2011). Rhizodephozition is a dynamic and extremely complex process, where there is a loss of reduced C for the plant and where the organic C pool enters the soil. This stimulates the biological activity in the rhizosphere (Jones et al. 2009). Microbial activity in soil is greatly influenced by the loss of carbon-containing metabolites where up to 40 % of photosynthetically fixed carbon is secreted into the rhizosphere by plant roots (Bais et al. 2006). In non-polluted soils, the higher C costs to plants of maintaining both fungal and bacterial symbionts may result in indirect antagonistic interactions between symbionts (Bethlenfalvay et al. 1982; Mortimer et al. 2008; Franzini et al. 2010). However, under unfavorable conditions, plants prefer co-inoculation strategy with AMF and N_2_-fixers.

Extracellular polysaccharides (EPS) were purposed by Bianciotto 2001 to play a crucial role in the anchoring of *A. brasilense* and *R. leguminosarum* and in the formation of biofilms on the root and the AM fungus (Bianciotto et al. 2001). Biofilm microorganisms may play an essential role as mediators in the transfer of heavy metals (García-Meza et al. 2005). Extracellular polymeric substances potentially act as detoxification agents against metals, acting as metal-binding sites. Biofilm occurrence seems to enhance complexation and immobilization of Cr, Ni, Cu, Zn, As, and Pb. Due to the organic matter improvement, the biofilms could also be conceptualized as an organic cover, which controls pH (Lukešová 2001; García-Meza et al. 2006). The persistence of the photosynthetically active forms such as cyanobacteria might result in a natural fertilization of the tailing substratum building up an appropriate environment for further plant establishment. The importance of soil cyanobacteria in increasing soil fertility, through the input of nitrogen, promotes the release of nutrients from insoluble compounds (Maxwell 1991). Studies conducted by Trzcińska and Pawlik-Skowrońska 2008 refer to the cyanobacteria isolated from Zn–Pb-loaded soils, including *N. edaphicum* strain. Those isolated strains are Zn-Pb-resistant ecotypes, which have been rarely reported, in other terrestrial environment (Trzcińska and Pawlik-Skowrońska 2008). *N. edaphicum* can utilize various inorganic and organic nitrogen sources for growth. Those nitrogen sources are utilized in the hierarchical order of NH4^+^ > NO^3−^ > N_2_. This flexibility allows *Nostocales* to colonize and compete as a phototroph in illuminated habitats, irrespective of the specific nitrogen source (Meeks et al. 2001). This ability seems to be important especially in poor nutrient environment such as waste tailing. The present studies reveal positive effect on plant growth by dual inoculation with native strain of *N. edaphicum* and AM fungi. This might be related to nitrogen level available for AMF symbionts. However, single inoculation with cyanobacteria or with nitrogen-fixing bacteria did not have a positive effect on plant growth. In both types of substrate treatments (S and NS), AMF colonization stimulated the formation of root nodules in the alfalfa roots. AMF colonization has been known to enhance the formation of nodules, by increase P which then attributed to better N uptake, because of higher nitrogenase-fixation activity in mycorrhizal plants (Toro et al. 1998; Andrade et al. 2004; Barea et al. 2005b; Lin et al. 2007). Also, this positive role of AMF might be related to biofilm formation. Bacteria living in the biofilm forms have the ability to survive much harsher environmental conditions (Costerton et al. 1995). On the other hand, dual symbiosis formed by AMF with *Rhizobium* strain has been shown to inhibit nodule development and N_2_-fixation of *Phaseolus vulgaris* (Lin et al. 2007; Franzini et al. 2010). Given this case that the formation of root nodules may be strongly inhibited not just by heavy metals present in the soil, but also by other microorganisms, therefore, a selection of appropriated symbionts to specific plants and environmental condition is needed to improve successful phytoremediation. This may be due to the competition for nutrients between mixed microorganisms and the ability of one organism to deal with heavy metals more than the other (Hudek et al. 2012).

### Plant photosynthesis

The JIP test is presently widely accepted for evaluation of PSII behavior. Organisms exposed to stress such as high light level or heat showed pronounced decrease of φPo = TR_0_/ABS, measured as F_v_/F_m_ (Strasser et al. 2004). Plants grown under optimal conditions show values of F_v_/F_m_ in the range 0.79–0.85 (Maxwell and Johnson 2000; Kalaji et al. 2014). Similar values were observed in case of plants studied in the present research. In comparison to control plants (grown alone without addition of inocula), microbial inoculation resulted in significant increase of F_v_/F_m_ parameter for *H. pilosella*, except the case when *H. pilosella* was only co-cropped with *M. sativa*. On non-sterilized substrate, F_v_/F_m_ values were significantly lower, only when triplicate co-inoculation was provided. Decrease of F_v_/F_m_ already suggests negative effect of inoculation on photosynthesis functioning. *M. sativa* did not exhibit any changes regarding F_v_/F_m_ parameters. Previous studies indicated that performance indexes (PI_TOTAL_, PI_ABS_) and driving forces (DF_TOTAL_, DF_ABS_) are sensitive measures of plant responses to different kinds of environmental stresses such as irradiance, drought, heat, salt, biotic-stressed, and exposure to heavy metals (Tsimilli-Michael et al. 2000; Strasser et al. 2000; Strauss et al. 2006; Christen et al. 2007; Yusuf et al. 2010; Kalaji et al. 2011; Oukarroum et al. 2014). Therefore, these parameters are consistent to evaluate the plant performance, especially when plants were exposed to the stress (Strasser et al. 2000; Strauss et al. 2006). Findings that microbes can cause a stress have been also reported earlier (Tsimilli-Michael et al. 2000; Tsimilli-Michael and Strasser 2002). Similarly to abovementioned parameters, the test showed differences in (ABS/RC), trapping (TR_0_/RC), and reduction of end acceptors at PSI electron acceptor side (ET_0_/RC) and the dissipated energy flux (DI_0_/RC) between controls and plants inoculated with microbes as was shown in Tsimilli-Michael et al. 2000 and Strasser et al. 2004. ABS/RC that gives the total absorption of chlorophylls in PSII antennae per reaction centers is a good measure for average functional antenna size (Tsimilli-Michael et al. 2000). Higher values of ABS/RC together with significant increase in DI_0_/RC observed in control plants (*M. sativa* and *H. pilosella*) could be considered as indicators of photoinhibitory damages to PSII complexes (Force et al. 2003). Co-cropping system that includes *M. sativa* and *H. pilosella* without microbial inoculation is not sufficient for successful remediation. Under laboratory conditions, plants are not exposed to such extreme stresses as the one observed on industrial wastes. Therefore, the chances for plant survival are even lower. The use of microbes visibly attenuates negative effect of co-cropping and additionally can be useful for nutrient cycling. Figures 6 and 5 indicate similar trends in plant behavior depending on the use of substrate sterilization procedure. Under sterile conditions, both plants exhibited higher PI parameters when triple inoculation was performed. If substrate was not sterilized, the addition of bacterial inoculation had even more negative effect on plant performance. As shown above, JIP test provides a useful tool for evaluation of the effectiveness of microbial inoculation that has to be taken into account, especially when phytoremediation of polluted site has to be optimized.

## Conclusions

Future bioremediation research should strive toward an improved understanding of the functional mechanisms behind such microbial interactions, so that optimized combinations of microorganisms can be applied as effective inoculants within sustainable remediation systems. Appropriate bioremediation practices may include these inoculates to obtain a beneficial effect on plant establishment on heavy metal-polluted sites. Our experimental tests confirm that both negative and positive feedbacks occur between plants and microbial communities. An improved understanding of the interactions taking place in the rhizosphere will help to translate the results of simplified experiments into field application. The characteristics of a microbial network in laboratory conditions could help to design specific remediation strategy on metal-rich industrial wastes.

## Electronic supplementary material

ESM 1 (DOCX 53 kb)
