# Supplementary material for: Effect of combined microbes on plant tolerance to Zn–Pb contaminations
Source: Environ Sci Pollut Res Int. 2015 Aug 7;22(23):19142–56. doi: 10.1007/s11356-015-5094-2 (PMC4669377; doi:10.1007/s11356-015-5094-2)
Supplement: Supplementary file 1 — (DOCX 53 kb) [file 11356_2015_5094_MOESM1_ESM.docx]

PCA analysis

- black- plants grown on non-sterile substrate (NS)
- red - plants grown on sterile substarte (S)

***Medicago sativa***

**Sterile (S) and Non-Sterile (NS)**

| Statistic | Axis 1 | Axis 2 | Axis 3 | Axis 4 | Axis 5 | Axis 6 |
| --- | --- | --- | --- | --- | --- | --- |
| Eigenvalues | 0.3997 | 0.2740 | 0.1331 | 0.0969 | 0.0346 | 0.0327 |
| Explained variation (cumulative) | 39.97 | 67.37 | 80.68 | 90.38 | 93.84 | 97.11 |

Loadings

| ALL | axis1 | axis2 | axis3 |
| --- | --- | --- | --- |
| t for Fm | 0,265 | 0,4485 | -0,0323 |
| Area | -0,2294 | 0,7574 | -0,3771 |
| Fo | 0,8236 | 0,2801 | -0,3216 |
| Fm | 0,2137 | -0,0344 | -0,9597 |
| Fv | 0,0124 | -0,1154 | -0,9777 |
| Fv/Fm | -0,7151 | -0,344 | -0,5008 |
| Fv/Fo | -0,7032 | -0,3212 | -0,5081 |
| Vj | 0,7501 | -0,3576 | -0,1016 |
| Vi | 0,4368 | -0,8341 | -0,1796 |
| dVG/dto | 0,7905 | 0,004 | -0,1486 |
| Sm | -0,2241 | 0,821 | 0,1779 |
| N | 0,2905 | 0,8965 | 0,0126 |
| Sm/t(Fm) | -0,3993 | -0,1359 | 0,1321 |
| ABS/RC | 0,8542 | 0,3286 | -0,1303 |
| DIo/RC | 0,8905 | 0,3944 | 0,1564 |
| TRo/RC | 0,7948 | 0,2856 | -0,2469 |
| ETo/RC | 0,3687 | 0,6539 | -0,236 |
| REo/RC | -0,0115 | 0,9071 | 0,0534 |
| phi(Po) | -0,7133 | -0,3413 | -0,503 |
| phi(Eo) | -0,8671 | 0,2355 | -0,0497 |
| delta(Ro) | -0,2216 | 0,8228 | 0,1735 |
| phi(Ro) | -0,5243 | 0,7868 | 0,1267 |
| DIo/CSo | 0,8478 | 0,3468 | 0,0841 |
| TRo/CSo | 0,7544 | 0,2248 | -0,5089 |
| ETo/CSo | 0,0802 | 0,5562 | -0,4421 |
| REo/CSo | -0,1716 | 0,9523 | -0,0256 |
| ETo/CSm | -0,5319 | 0,1674 | -0,7847 |
| REo/CSm | -0,4835 | 0,7773 | -0,2521 |
| PI abs | -0,96 | 0,0172 | -0,0308 |
| PI total | -0,8426 | 0,5031 | 0,0789 |
| DF abs | -0,9575 | -0,0082 | -0,0488 |
| DF Total | -0,8243 | 0,4556 | 0,0692 |

***Hieracium pilosella***

**Sterile (S) and Non-Sterile (NS)**

| Statistic | Axis 1 | Axis 2 | Axis 3 | Axis 4 | Axis 5 | Axis 6 |
| --- | --- | --- | --- | --- | --- | --- |
| Eigenvalues | 0.4576 | 0.2581 | 0.2018 | 0.0483 | 0.0140 | 0.0084 |
| Explained variation (cumulative) | 45.76 | 71.58 | 91.75 | 96.58 | 97.97 | 98.81 |
|  |  |  |  |  |  |  |

1 i 2 oś 1 i 3 oś

Loadings:

| ALL | axis1 | axis2 | axis3 |
| --- | --- | --- | --- |
| t for Fm | 0,3483 | -0,2583 | 0,6429 |
| Area | 0,4151 | -0,887 | -0,1099 |
| Fo | 0,6367 | -0,6581 | -0,3845 |
| Fm | 0,2826 | -0,7251 | -0,6257 |
| Fv | 0,1739 | -0,717 | -0,672 |
| Fv/Fm | -0,8197 | -0,0851 | -0,5083 |
| Fv/Fo | -0,8147 | -0,107 | -0,5011 |
| Vj | 0,6531 | 0,4885 | -0,4979 |
| Vi | -0,3898 | 0,3969 | -0,7985 |
| dVG/dto | 0,7843 | 0,4592 | -0,2789 |
| Sm | 0,4225 | -0,4153 | 0,762 |
| N | 0,8583 | -0,1395 | 0,4227 |
| Sm/t(Fm) | -0,1829 | -0,0587 | -0,2464 |
| ABS/RC | 0,9041 | 0,337 | -0,2015 |
| DIo/RC | 0,9599 | 0,2048 | 0,1434 |
| TRo/RC | 0,8229 | 0,3749 | -0,3539 |
| ETo/RC | 0,8686 | 0,2167 | -0,2178 |
| REo/RC | 0,8765 | -0,1223 | 0,3649 |
| phi(Po) | -0,8194 | -0,0848 | -0,5084 |
| phi(Eo) | -0,8559 | -0,4425 | 0,1746 |
| delta(Ro) | 0,6607 | -0,2561 | 0,6452 |
| phi(Ro) | 0,1854 | -0,4937 | 0,7903 |
| DIo/CSo | 0,8299 | -0,5103 | -0,1305 |
| TRo/CSo | 0,5513 | -0,6883 | -0,4615 |
| ETo/CSo | 0,4122 | -0,8289 | -0,3576 |
| REo/CSo | 0,6118 | -0,7851 | -0,0039 |
| ETo/CSm | 0,0286 | -0,8285 | -0,5574 |
| REo/CSm | 0,3289 | -0,9086 | -0,2158 |
| PI abs | -0,9125 | -0,3767 | 0,1055 |
| PI total | -0,6562 | -0,5254 | 0,4968 |
| DF abs | -0,927 | -0,3564 | 0,0753 |
| DF Total | -0,6513 | -0,5333 | 0,4755 |

*t for Fm:* time (in ms) to reach Fm

*Area:* area above the OJIP curve between F _0_ and F_m_ and the F_m_ asymptote

*F _O_ :*minimum Chl *a* fluorescence yield in the dark-adapted state

*F _M_ :*maximum Chl *a* fluorescence yield in the dark-adapted state

*F _V_/F _M_ :*a quantity related to the maximum quantum yield of PSII photochemistry

*F _O_/F _M_ :*a parameter related to changes in heat disipation in the photosystem II antenna

*Vj:* relative variable Chl a fluorescence at the J-step

*Vi:* relative variable Chl a fluorescence at the I-step

*dVG/dto:* an approximate initial slope (in ms−1 ) of the fluorescence transient

*Sm:*normalized area above the OJIP transient

*N:*non-photochemical quenching, expressed as (1 − *F* _V_′/*F* _V_)

*Sm/t(Fm):* normalized total complementary area above the OJIP transient (reflecting multipleturnover

*Q*A reduction events) or total electron carriers per RC

***Specific fluxes or activities per reaction center (RC)***

*ABS/RC:* the average absorption per RC

*DI_0_/RC:* the dissipated energy flux per RC

*TR_0_/RC:* the specific trapping flux per RC

*ET_0_/RC:* the maximal specific flux for electron transport per RC

***Yields or flux ratios***

*φ Po = TRo/ABS = 1 – Fo/Fm = Fv/Fm:* maximum quantum yield of primary photochemistry (at *t* = 0)

*φ Eo= ETo/ABS = (Fv/Fm)(1 – VJ):* quantum yield of electron transport (at *t* = 0)

*δ Ro= REo/ETo = (1 – VI)/(1 – VJ):* efficiency/probability with which an electron from the intersystem electron carriers moves to reduce end electron acceptors at the PSI acceptor side (RE)

*φ Ro= REo/TRo = ψEo δRo:* quantum yield for reduction of end electron acceptors at the PSI acceptor side (RE)

***Phenomenological fluxes or activities per excited cross section***

*DIo/CSo = ABS/CSo – TRo/CSo:* dissipated energy flux per CS at *t* = 0

*TRo/CSo = (ABS/CSo)ϕPo*: trapped energy flux per CS at *t* = 0

*ETo/CSo = (ABS/CSo)ϕEo:* electron transport flux per CS at *t* = 0

*REo/CSo = (REo/ETo)(ETo/CSo):* reduction of end acceptors at PSI electron acceptor side per CS at *t* = 0

*ETo/CSm:* electron transport flux from reduced QA to QB per excited cross-section CSm

*REo/CSm:* electron transport flux from reduced QB to PSI end acceptors per excited cross-section CSm

***Performance index***

*PI_ABS_:* Performance index (PI) on absorption basis

*PI_TOTAL_:* Total PI, measuring the performance up to the PSI end electron acceptors

***Driving force***

*DF_ABS:_* driving force on absorption basis

*DF_TOTAL_* total driving force
